# The diagnostic value of CT-based radiomics nomogram for solitary indeterminate smoothly marginated solid pulmonary nodules

**DOI:** 10.3389/fonc.2024.1427404

**Published:** 2024-07-02

**Authors:** Chengzhou Zhang, Huihui Zhou, Mengfei Li, Xinyu Yang, Jinling Liu, Zhengjun Dai, Heng Ma, Ping Wang

**Affiliations:** ^1^ Department of Radiology, Yantai Yuhuangding Hospital, Affiliated Hospital of Qingdao University, Yantai, Shandong, China; ^2^ Department of Pathology, Yantai Yuhuangding Hospital, Affiliated Hospital of Qingdao University, Yantai, Shandong, China; ^3^ School of Medical Imaging, Binzhou Medical University, Yantai, Shandong, China; ^4^ Scientific Research Department, Huiying Medical Technology Co., Ltd, Beijing, China

**Keywords:** lung, nodule, computed tomography, radiomics, nomogram

## Abstract

**Objectives:**

This study aimed to explore the value of radiomics nomogram based on computed tomography (CT) on the diagnosis of benign and malignant solitary indeterminate smoothly marginated solid pulmonary nodules (SMSPNs).

**Methods:**

This study retrospectively reviewed 205 cases with solitary indeterminate SMSPNs on CT, including 112 cases of benign nodules and 93 cases of malignant nodules. They were divided into training (n=143) and validation (n=62) cohorts based on different CT scanners. Radiomics features of the nodules were extracted from the lung window CT images. The variance threshold method, SelectKBest, and least absolute shrinkage and selection operator were used to select the key radiomics features to construct the rad-score. Through multivariate logistic regression analysis, a nomogram was built by combining rad-score, clinical factors, and CT features. The nomogram performance was evaluated by the area under the receiver operating characteristic curve (AUC).

**Results:**

A total of 19 radiomics features were selected to construct the rad-score, and the nomogram was constructed by the rad-score, one clinical factor (history of malignant tumor), and three CT features (including calcification, pleural retraction, and lobulation). The nomogram performed better than the radiomics model, clinical model, and experienced radiologists who specialized in thoracic radiology for nodule diagnosis. The AUC values of the nomogram were 0.942 in the training cohort and 0.933 in the validation cohort. The calibration curve and decision curve showed that the nomogram demonstrated good consistency and clinical applicability.

**Conclusion:**

The CT-based radiomics nomogram achieved high efficiency in the preoperative diagnosis of solitary indeterminate SMSPNs, and it is of great significance in guiding clinical decision-making.

## Introduction

Pulmonary nodules are common in clinical practice, and the correct differentiation between benign and malignant nodules is critical in guiding treatment planning. Pulmonary nodules can be classified into subsolid and solid nodules based on computed tomography (CT) density (1–4). Solid nodules have proven to be more difficult to make a correct diagnosis than subsolid nodules. Studies on patients undergoing surgical resection have shown that over 95% of subsolid nodules are malignant, whereas the malignant rate of solid nodules ranges from 51% to 67% (4–6). In addition, the malignant degree of solid lung cancers is higher than that of subsolid ones, which are not suitable for long-term follow-up (1, 2, 7). Therefore, the accurate and timely diagnosis of solid nodules must be improved.

Spiculation sign is defined as the presence of strands radiating from the margin of the nodule to the lung parenchyma without reaching the surface of the pleura (8). Spiculation sign is a well-known sign associated with malignancy (3–5, 8, 9). Studies have found that benign solid nodules usually present with a well-defined smooth margin, whereas most malignant solid nodules show spiculated and ill-defined margins (3, 10–12). In clinical practice, solitary smoothly marginated solid pulmonary nodules (SMSPNs) tend to be diagnosed as benign. However, we found that many malignant nodules present with similar characteristics. For SMSPNs, the presence of fat and/or benign calcification pattern (including central, diffuse, laminated, and “popcornlike”) within the nodules are highly specific indicators of benignancy (10, 11). In addition, the risk of lung cancer of smoothly marginated triangular, lentiform, oval, or semicircular juxtapleural nodules is extremely low (10, 12–14). Except for the above situations, differentiating benign from malignant SMSPNs by CT features is usually difficult. Thus, new methods are needed to improve the diagnostic ability for indeterminate SMSPNs.

Radiomics is a research hotspot in recent years. It can extract a large number of high-dimensional and quantifiable features from traditional CT images, as well as combine clinical factors and traditional CT features to establish prediction models via artificial intelligence methods, thereby achieving disease diagnosis, lymph node metastasis, and prognosis prediction (5, 6, 9, 15–17). We hypothesized that the combination of additional radiomics features within the nodules can improve the diagnostic ability for indeterminate SMSPNs. Therefore, we constructed a nomogram that combined the CT-based radiomics features, clinical factors, and conventional CT features for the diagnosis of benign and malignant solitary indeterminate SMSPNs.

## Materials and methods

### Patients

Patients with lung nodule who underwent surgical resection at our institution were retrospectively analyzed from February 2019 to March 2022. Our Institutional Ethics Committee approved this study. Because this was a retrospective study, the requirement for written informed consent was waived. The inclusion criteria were as follows: nodule size ≤ 3 cm, solid nodule on CT, lesion was located away from the segmental bronchi, and nodule had a well-defined smooth edge. The exclusion criteria were as follows: nodule contained fat, nodule contained benign calcification (including central, diffuse, laminated, and “popcornlike”), juxtapleural nodule, and lesion received chemotherapy or radiotherapy prior to surgery.

Data on clinicopathological characteristics, including sex, age, history of malignant tumor, smoking history, serum preoperative carcinoembryonic antigen (CEA) level, cytokeratin 19 fragments (CYFRA) level, neuron-specific enolase (NSE) level, squamous cell carcinoma antigen (SCCA) level, and postoperative pathology of nodule were collected.

### CT technique

Chest CT was conducted by multislice spiral CT scanners, including dual-source Somatom Force 256, SOMATOM Definition AS 128 (Siemens, Germany), iCT 256, Brilliance 64 (PHLIPS, Holland), or Light Speed 64 (GE, USA). The scanning parameters were 120 kVp, 120–250 mAs; slice thickness, 5 mm; reconstruction thickness of 1 mm or 1.25 mm; matrix, 512 mm × 512 mm; lung window width/level, 1500/–550 Hounsfield unit (HU); and mediastinum window width/level, 350/40 HU.

### CT feature interpretation

Two radiologists who specialized in thoracic radiology (one with 13 years and one with 15 years of work experience) reviewed all the CT images independently. They were blinded to the histopathological data and resolved disagreements by consensus.

The evaluated CT signs included (1) nodule size (the largest diameter of nodule measured on the lung window image), (2) CT attenuation value (HU), (3) location (right upper lobe, right middle lobe, right lower lobe, left upper lobe, or left lower lobe), (4) lobulation, (5) bronchial cut-off, (6) pleural retraction, (7) calcification [other types besides benign calcification pattern (including central, diffuse, laminated, and “popcornlike”)], and (8) lymph node enlargement (the short axial diameter ≥ 1 cm on CT).

The Intraclass Correlation Coefficient (ICC) and Kappa statistics were utilized to assess interobserver agreement in CT feature interpretation. The ICC was employed for quantitative data analysis, while Kappa statistic was utilized for categorical data analysis. Finally, the above two radiologists classified all nodules as benign or malignant based on preoperative clinical factors and CT images.

### Radiomics feature extraction

The latest guidelines set forth by the Image Biomarker Standardization Initiative (IBSI) were meticulously adhered to in the analysis of radiomics features (18). Specifically, feature extraction was performed on a RadCloud platform (version 7.2, Huiying Medical Technology Co., Ltd, Beijing, China, https://mics.huiyihuiying.com) (19). The platform is based on the IBSI-compliant PyRadiomics (20). The region of interest (ROI) was delineated on axial thin-section lung window images by a semi-automatic delineation method.

In our study, gray level discretization was executed with a consistent bin width of 25, and voxel sizes were resampled to a uniform dimension of 1mm×1mm×1mm using the PyRadiomics software package. For each ROI, 1688 radiomics features were extracted in our study. In each three-dimensional segmentation, a total of 107 radiomics features were extracted from the original image, including 14 three-dimensional shape features, 18 first-order statistics features, 14 Gray Level Dependence Matrix features, 16 Gray Level Size Zone Matrix features, 16 Gray Level Run Length Matrix features, 24 Gray Level Co-occurrence Matrix features, and 5 Neighboring Gray Tone Difference Matrix features. Following this, texture characteristics were derived from filtered images including Square, Square Root, Wavelet, Exponential, Logarithm, Gradient, two-dimensional local binary pattern, and three-dimensional local binary pattern.

### Radiomics feature selection and Rad-score construction

First, 50 patients were randomly selected for intra- and interobserver reproducibility of feature selection. The specific step was that the ROI of the 50 patients’ images was separately delineated by two radiologists, and the features were extracted. After 1 month, the ROI of the 50 patients’ images was delineated again by the junior radiologist, and the features were extracted. The intra- and interobserver reproducibility of feature selection was tested by ICC, and features with ICC value greater than 0.75 were selected for further feature analysis. Subsequently, the ROI of the rest patients’ images was delineated by the junior radiologist. The variance threshold method, SelectKBest, and least absolute shrinkage and selection operator (LASSO) were used to select the key radiomics features to construct the rad-score.

### Nomogram building and validation

After univariate and multivariate logistic regression analyses, a nomogram was constructed by combining the rad-score, clinical factors, and CT features, which were significantly different (P<0.05) between benign and malignant nodules in the training cohort. The nomogram performance was verified by area under the receiver operating characteristic curve (AUC) value, sensitivity, specificity, accuracy, calibration curve, and clinical decision curve analysis. In addition, the diagnosis ability of the nomogram was compared with the radiomics model (rad-score), the clinical model (constructed by CT features and clinical factors), and radiologists’ diagnosis.

### Statistical analysis

SPSS software (Version 23.0) and R software (version 3.4.2) were used for statistical analysis. χ2 or Fisher’s exact test were used for categorical variables, while the Mann–Whitney U test or two-sample t-test were used for continuous variables. The AUC values among the models were compared via DeLong test. P value < 0.05 indicated statistical significance.

## Results

### Patients’ characteristics and conventional CT features

**Figure 1:**
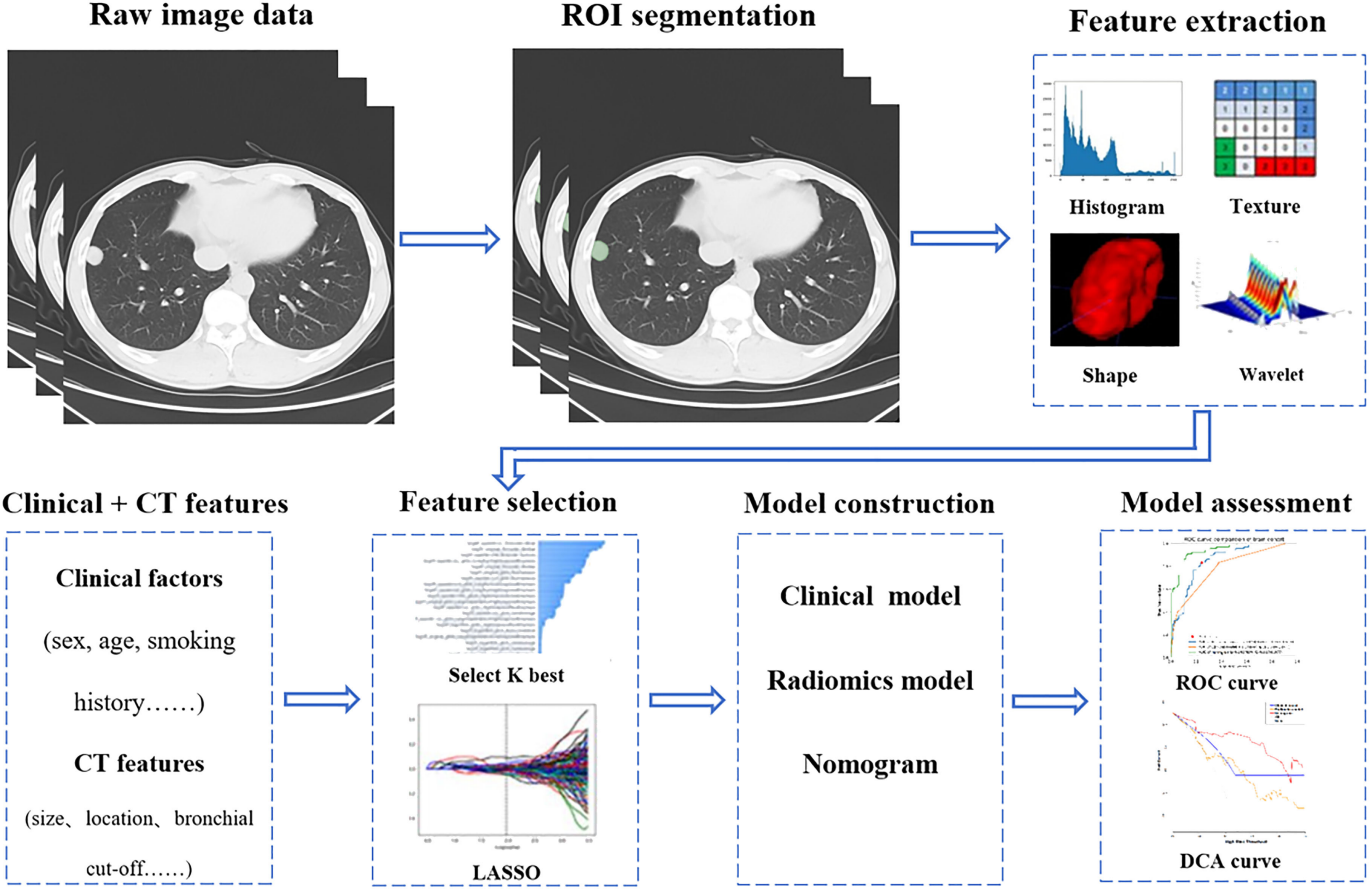
Flow chart of the study.

The study flow chart is presented in **Figure 1**. In the end, 205 patients were enrolled in this study. Among them, 112 patients had benign nodules, whereas 93 patients had malignant nodules. The most common benign nodules were hamartomas (n=61), followed by inflammatory nodules (n=39), sclerosing pulmonary cell tumors (n=10), ciliated mucinous nodular papillary tumor (n=1), and alveolar adenoma (n=1). The most common malignant nodules were adenocarcinomas (n=34), followed by metastatic tumors (n=29), small cell lung cancers (n=19), and squamous cell carcinomas (n=11).

Among the 205 patients, 143 (scanned at CT 1, 2, and 3) were selected as the training cohort, and 62 (scanned at CT 4 and 5) were selected as the validation cohort. The patients’ clinical factors and CT features are summarized in **Table 1**.

**Table 1:** The patients’ characteristics and conventional CT features of the training and validation cohorts.

Characteristics	Training cohort			Validation cohort		
	Benign nodule (n=78)	Malignant nodule (n=65)	*P* value	Benign nodule (n=34)	Malignant nodule (n=28)	*P* value
Sex (%)						
Male	32/78	39/65	0.024	14/34	17/28	0.126
Female	46/78	26/65		20/34	11/28	
**Age (year)**	56.4 ± 10.6	58.8 ± 10.2	0.182	56.5 ± 10.3	60.8 ± 10.6	0.110
History of malignant tumor (%)						
Absent	74/78	44/65	<0.001	33/34	21/28	0.018
Present	4/78	21/65		1/34	7/28	
Smoking history (%)						
Absent	70/78	50/65	0.038	26/34	19/28	0.449
Present	8/78	15/65		8/34	9/28	
CEA level (ng/mL) (%)						
≤ 5	76/78	59/65	0.141	33/34	27/28	1
> 5	2/78	6/65		1/34	1/28	
CYFRA level (ng/mL) (%)						
≤ 3.3	63/78	54/65	0.722	26/34	23/28	0.585
> 3.3	15/78	11/65		8/34	5/28	
NSE level (ng/mL) (%)						
≤ 17	68/78	55/65	0.660	29/34	22/28	0.523
> 17	10/78	10/65		5/34	6/28	
SCCA level (ng/mL) (%)						
≤ 1.5	77/78	60/65	0.092	33/34	28/28	1
> 1.5	1/78	5/65		1/34	0/28	
**Size (mm)**	10.5 (5.8–13.7)	12.3 (6.3–16.4)	0.223	9.1 (6.0–11.4)	15.1 (10.2–19.3)	< 0.001
**CT attenuation value (HU)**	18.4 (12.3–25.6)	20 (12–32)	0.579	15.6 (6.3–21.9)	19.5 (13.2–24.1)	0.159
Location (%)						
Right upper lobe	18/78	13/65	0.065	7/34	4/28	0.686
Right middle lobe	13/78	7/65		1/34	3/28	
Right lower lobe	15/78	10/65		9/34	9/28	
Left upper lobe	7/78	18/65		8/34	7/28	
Left lower lobe	25/78	17/65		9/34	5/28	
Lymph node enlargement (%)						
Absent	78/78	59/65	0.008	34/34	22/28	0.006
Present	0/78	6/65		0/34	6/28	
Lobulation (%)						
Absent	45/78	20/65	0.001	10/34	6/28	0.475
Present	33/78	45/65		24/34	22/28	
Bronchial cut-off (%)						
Absent	71/78	46/65	0.002	33/34	22/28	0.039
Present	7/78	19/65		1/34	6/28	
Pleural retraction (%)						
Absent	76/78	55/65	0.006	34/34	24/28	0.037
Present	2/78	10/65		0/34	4/28	
Calcification (%)						
Absent	63/78	61/65	0.022	25/34	28/28	0.003
Present	15/78	4/65		9/34	0/28	

CEA, Carcinoembryonic antigen; CYFRA, Cytokeratin 19 fragments; NSE, Neuron-specific enolase; SCCA, Squamous cell carcinoma antigen; HU, Hounsfield unit.

The consistency in interpretation of CT features among radiologists demonstrated good interobserver agreement, with ICC values ranging from 0.913 to 0.953, and Kappa values ranging from 0.798 to 0.962.

### Radiomics feature selection and Rad-score construction

**Figure 2:**
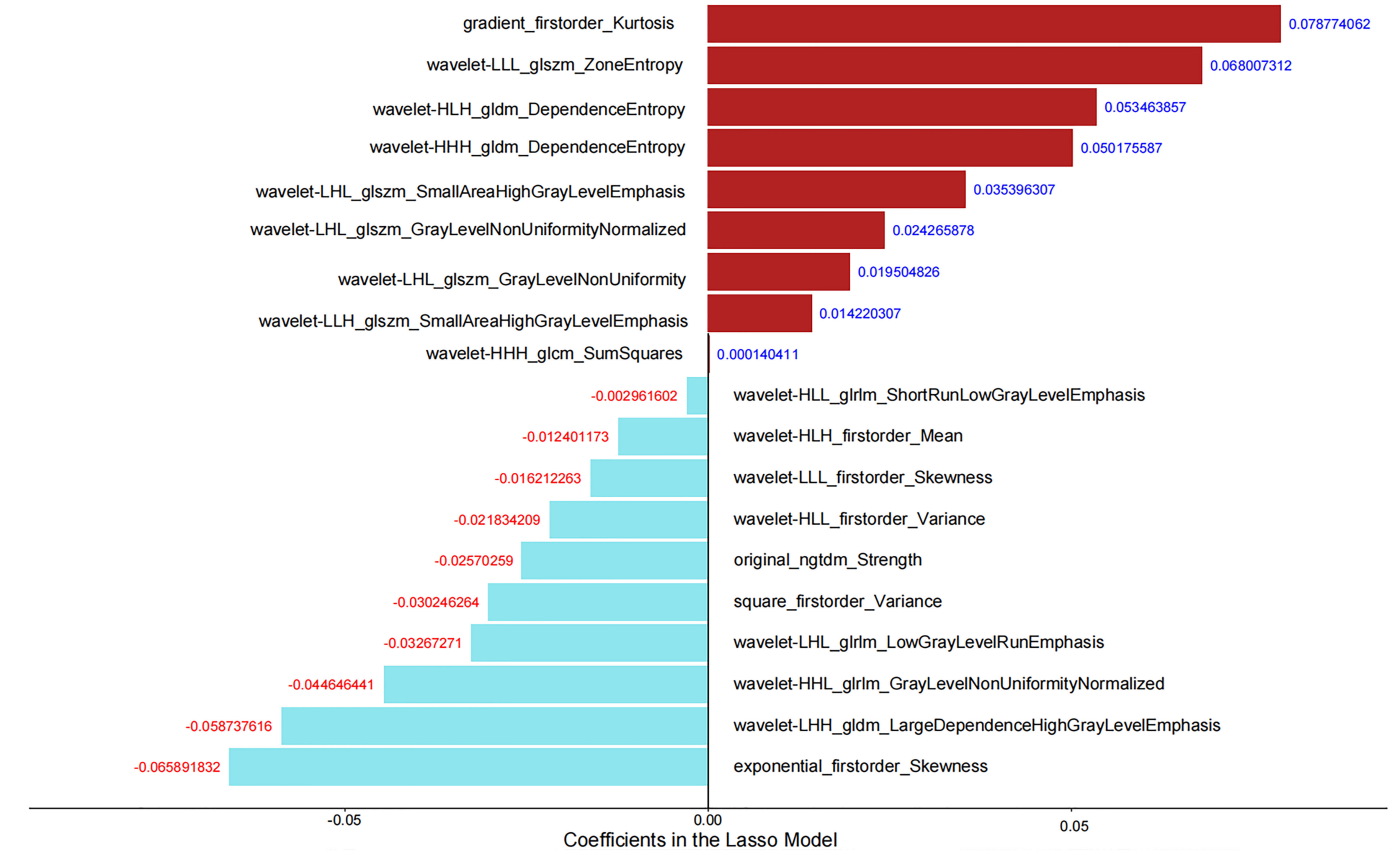
Nineteen key radiomics features and corresponding coefficients.

A total of 1688 radiomics features were extracted in this study, and 1565 features remained after excluding those with ICC values less than 0.75 in intra- and interobserver reproducibility. Using the variance threshold, which was set to 0.8, 1234 features were selected. A total of 232 features were obtained via SelectKBest with P value < 0.05. Finally, 19 features were left via the LASSO algorithm with five-fold cross-validation (**Figure 2**). The rad-score was constructed by the 19 key radiomics features.

### Nomogram building

**Figure 3:**
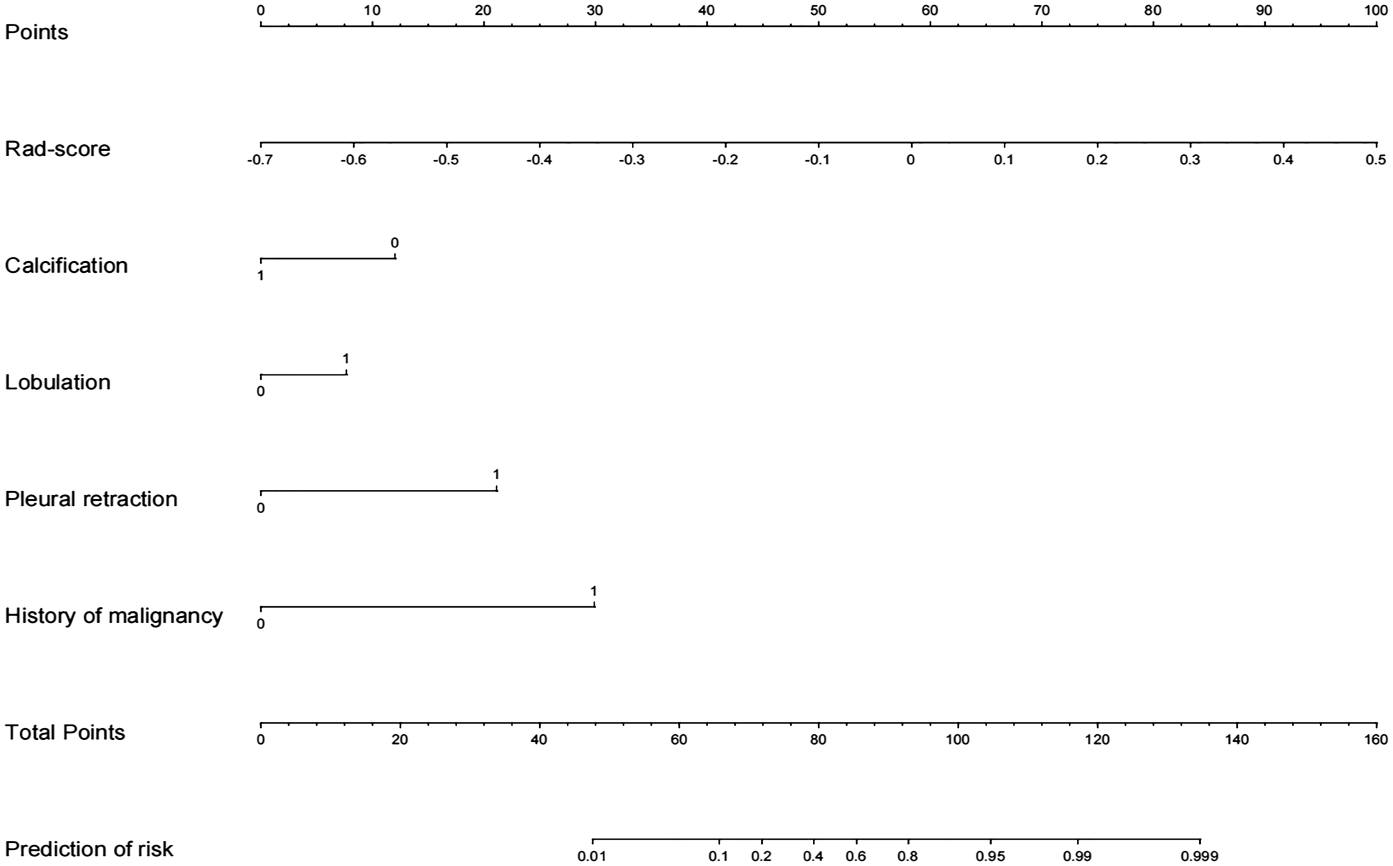
Radiomics nomogram with rad-score, calcification, lobulation, pleural retraction, and history of malignant tumor.

After univariate and multivariate logistic regression analyses, the rad-score, one clinical factor (history of malignant tumor), and three CT features (namely, calcification, pleural retraction, and lobulation) were identified as independent factors for the diagnosis of indeterminate SMSPNs (**Table 2**). The nomogram was constructed using the above selected factors (**Figure 3**). The P value of the Hosmer–Lemeshow test was 0.706, which indicated the good fit of the model.

**Table 2:** The independent clinical factors and conventional CT features for the diagnosis of indeterminate smoothly marginated solid pulmonary nodules.

Characteristics	Benign nodules	n	Malignant nodules	n
History of malignancy				
	Hamartoma	3/61	Metastasis	24/29
	Sclerosing pulmonary cell tumor	1/10	Adenocarcinoma	2/34
	Inflammatory nodule	1/39	Small cell lung cancer	1/19
	Ciliated mucinous nodular papillary tumor	0/1	Squamous cell carcinoma	1/11
	Alveolar adenoma	0/1		
Calcification				
	Hamartoma	19/61	Squamous cell carcinoma	2/11
	Inflammatory nodule	3/39	Adenocarcinoma	2/34
	Sclerosing pulmonary cell tumor	2/10	Metastasis	0/29
	Ciliated mucinous nodular papillary tumor	0/1	Small cell lung cancer	0/19
	Alveolar adenoma	0/1		
Pleural retraction				
	Hamartoma	1/61	Adenocarcinoma	14/34
	Inflammatory nodule	1/39	Metastasis	0/29
	Sclerosing pulmonary cell tumor	0/10	Small cell lung cancer	0/19
	Ciliated mucinous nodular papillary tumor	0/1	Squamous cell carcinoma	0/11
	Alveolar adenoma	0/1		
Lobulation				
	Hamartoma	41/61	Adenocarcinoma	26/34
	Inflammatory nodule	13/39	Squamous cell carcinoma	16/19
	Sclerosing pulmonary cell tumor	3/10	Metastasis	14/29
	Ciliated mucinous nodular papillary tumor	0/1	Squamous cell carcinoma	11/11
	Alveolar adenoma	0/1		

### Nomogram performance

**Figure 4:**
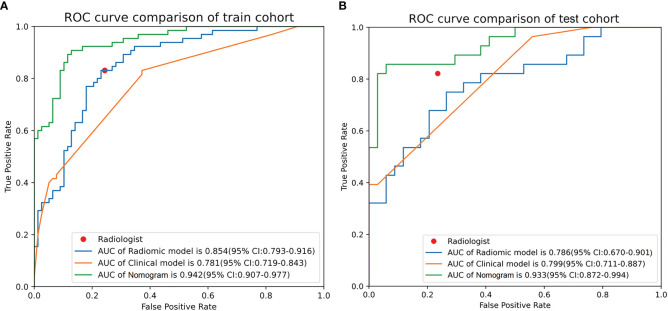
Receiver operating characteristic curves of nomogram, radiomics model, clinical model, and radiologists’ performance in the training **(A)** and validation **(B)** cohorts.

**Figure 5:**
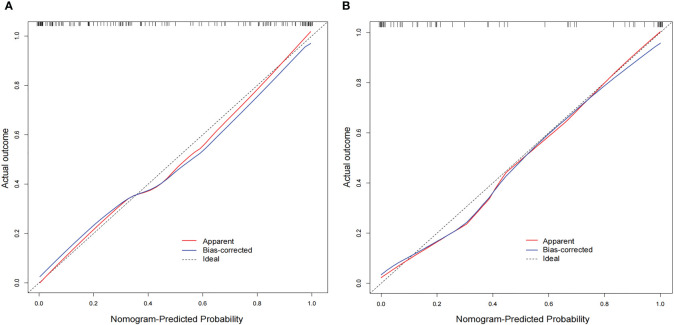
Calibration curves of the nomogram in the training **(A)** and validation **(B)** cohorts. The diagonal line is the reference line, which represents the “ideal” prediction. The red line represents the calibration curve of the nomogram, and the blue line indicates the correction bias of the nomogram. The calibration curves are close to the diagonal line, which indicated good prediction performance of the nomogram.

**Figure 6:**
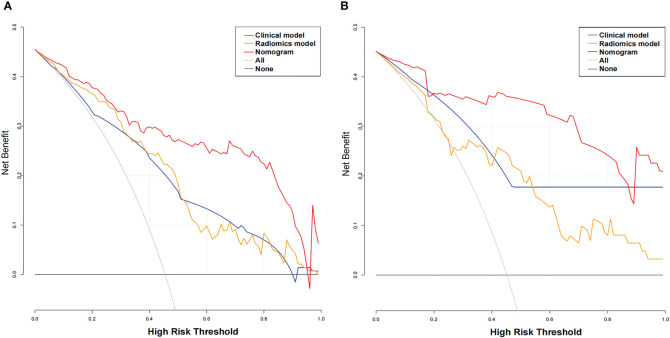
Decision curve analysis (DCA) for the three models. The x-axis indicates the threshold probability, whereas the y-axis indicates the net benefits. DCA showed that the net benefits of the nomogram (red line) were superior to those of the clinical model (blue line) and the radiomics model (orange line), with the threshold probability ranging from 0 to 0.96 in the training cohort **(A)** and from 0 to 0.9 in the validation cohort **(B)**.

The AUC values of the combined radiomics nomogram, radiomics model, and clinical model for the diagnosis of indeterminate SMSPNs were 0.942, 0.854, and 0.781, respectively, in the training cohort, and 0.933, 0.786, and 0.799, respectively, in the validation cohort. Delong test indicated that the AUC value of the nomogram was significantly higher than that of the radiomics model and clinical model in the training and validation cohorts (P<0.01), but no significant difference was observed in the AUC value between the radiomics model and clinical model in the training (P = 0.193) and validation cohorts (P = 0.784). The AUC values diagnosed by the radiologists were lower than those in the nomogram in the training and validation cohorts (**Figure 4**). The accuracy of the nomogram for nodule diagnosis was higher than that of the radiomics model, clinical model, and radiologists’ diagnosis in the training and validation cohorts (**Table 3**). The calibration curve (**Figure 5**) and decision curve (**Figure 6**) showed that the nomogram demonstrated good consistency and clinical applicability.

**Table 3:** Predictive performances of radiomics nomogram, radiomics model, clinical model, and radiologist’s judgment in the training and validation cohorts.

Model	Training cohort				Validation cohort			
	AUC (95% CI)	Sensitivity (95% CI)	Specificity (95% CI)	Accuracy	AUC (95% CI)	Sensitivity (95% CI)	Specificity (95% CI)	Accuracy
Radiomics nomogram	0.942 (0.907–0.977)	0.785	0.910	0.853	0.933 (0.872–0.994)	0.857	0.824	0.839
Radiomics model	0.854 (0.793–0.916)	0.785	0.795	0.790	0.786 (0.670–0.901)	0.750	0.735	0.742
Clinical model	0.781 (0.719–0.843)	0.815	0.628	0.713	0.799 (0.711–0.887)	0.964	0.441	0.677
Radiologist	NA	0.815	0.756	0.783	NA	0.788	0.794	0.790

AUC, Area under the receiver operating characteristic curve; CI, Confidence interval; NA, Not applicable.

## Discussion

Solitary SMSPNs are common in clinical practice, and many of such nodules are indeterminate by conventional CT features. In this study, we developed a CT-based nomogram incorporating the rad-score, clinical factors, and conventional CT features for the diagnosis of such nodules. The AUC values of the nomogram in the training and the validation cohorts were 0.942 and 0.933, respectively. These values showed that the nomogram performed better than the radiomics model, clinical model, and experienced radiologists who specialized in thoracic radiology. The calibration curve and decision curve showed that the nomogram demonstrated good consistency and clinical applicability.

Our study showed that a history of malignant tumor was an independent risk factor for malignancy in indeterminate SMSPNs (P < 0.05). In this study, among the 33 cases with a history of malignant tumor, malignant nodules were seen in 28 (84.8%) cases and benign nodules were seen in 5 (15.2%) cases. Metastasis was the most common pathological type, with up to 24 cases. The reason is related to the susceptibility of malignant tumors to lung metastasis, and lung metastases typically exhibit smooth margin on CT (21, 22). Therefore, for indeterminate SMSPNs, a history of malignant tumor strongly indicates a high likelihood of malignancy, especially metastases, even if they are solitary. Although gender and smoking history were significantly different between benign and malignant nodules in this study, multivariate logistic regression analysis showed no significant difference between the two groups.

The calcification pattern in the nodule is helpful for nodule diagnosis (5, 10, 11). Benign calcification comprises four patterns: central, diffuse, laminated, and “popcornlike.” The first three patterns are typically seen in chronic inflammatory nodules, whereas “popcornlike” calcifications are characteristic of hamartoma. Other calcification patterns include eccentric, punctate, stippled, and amorphous, which can be seen in benign and malignant nodules. Although this study excluded the nodules with benign calcification patterns, we found that the presence of other calcification patterns within the indeterminate SMSPNs was still an independent predictor for benign nodules (P < 0.05). In this study, a total of 28 nodules had calcification, of which 24 were benign nodules and only 4 were malignant nodules. The most common pathological type of calcified nodules was hamartoma, with up to 19 cases. Other calcified nodules included 3 inflammatory nodules, 2 squamous cell carcinomas, 2 adenocarcinomas, and 2 sclerosing pulmonary cell carcinomas. Calcification was most commonly seen in hamartoma due to the following reasons. First, hamartoma is mainly composed of cartilage, which exhibits variable degrees of calcification and ossification (23). Second, hamartoma is the most common pathological type of solitary indeterminate SMSPNs in this study, which was found in 61 cases.

Research has found that malignant nodules are more prone to have pleural retraction than benign nodules (5, 6, 24). In this study, pleural retraction was found to be an independent risk factor for malignancy in indeterminate SMSPNs (P < 0.05). Pleural retraction was identified in 16 nodules, among which the most common pathological type was adenocarcinoma in 14 cases, and the others included 1 inflammatory nodule and 1 hamartoma. The pleural retraction observed in adenocarcinoma is most likely related to the epithelial–mesenchymal transition. Some studies have found that the epithelial–mesenchymal transition in lung cancer is most likely to occur in adenocarcinoma; solid adenocarcinomas are more likely than subsolid adenocarcinomas to have epithelial–mesenchymal transition, which can lead to contractile force, pulling the pleura and causing pleural retraction (25, 26). Notably, all the nodules in this study were smoothly marginated without spiculation, and the pleural retraction was very mild or just appeared as a slight tension on the interlobular fissure pleura. Therefore, a combination of 3D reconstruction thin-layer images is required for careful observation of pleural retraction.

Lobulation was defined as an abrupt bulging of the contour of the lesion. Many studies have found that the lobulation sign is more commonly seen in malignant nodules than in benign nodules (3, 8, 17, 24). Similar to previous findings, our study found that lobulation sign was identified in 71.3% of malignant nodules versus 53.2% of benign nodules (P < 0.05), and lobulation sign was an independent risk factor for malignancy in SMSPNs (P < 0.05). It should be noted that a high proportion of hamartomas (67.2%) showed lobulation sign in this study. Hamartoma is mainly composed of cartilage, which is arranged in lobules separated by cleft-like branching channels and cystic spaces lined by respiratory epithelium, leading to lobulation sign on CT (23, 27, 28). In this study, univariate analysis showed that the probability of mediastinal lymph node enlargement and bronchial truncation sign was significantly higher in malignant nodules than in benign nodules (P < 0.05). However, multivariate logistic regression analysis showed that these factors were not independent factors for the diagnosis of indeterminate SMSPNs.

In addition to a detailed study of clinical factors and traditional CT signs, this study also investigated radiomics features extracted from the CT image of the nodule. This study extracted a total of 1688 radiomics features, and 19 key radiomics features were retained after feature selection. Among the 19 features, 18 were high-order statistical features. In addition, there were 14 texture features and 5 first-order features, without shape-based features. These results indicated that the intensity information and the relationship between pixels of the higher-order radiomics features within the nodules were meaningful for indeterminate SMSPNs diagnosis. The nomogram that incorporated rad-score, clinical factors, and CT features achieved better diagnostic efficiency than the radiomics model, clinical model, and experienced radiologists who specialized in thoracic radiology, which confirmed our hypotheses that the combination of additional radiomics features can improve the diagnostic ability for indeterminate SMSPNs.

At present, most research on the diagnosis of pulmonary nodules focused on differentiating between benign and malignant nodules, with some studies on different pathological subtypes, such as adenocarcinoma versus tuberculosis, or lung cancer versus organized pneumonia (5, 6, 9, 15, 17). With the exception of juxtapleural nodules and nodules with benign calcification or fat, most solitary SMSPNs are difficult to diagnosis by conventional CT features (10–14). Among the 205 indeterminate SMSPNs in this study, 111 (54.1%) were benign and 94 (45.9%) were malignant. A high proportion of benign nodules underwent unnecessary surgery, because of the low confidence in the diagnosis of such nodules by traditional CT. In clinical practice, solitary SMSPNs on CT tend to be diagnosed as benign; however, 45.9% of such nodules were malignant in this study. Misdiagnosis of malignant nodules as benign may result in uncontrolled tumor progression and poor prognosis. Therefore, a systematic study was carried out to differentiate benign from malignant indeterminate SMSPNs in this study. We found that a history of malignant tumor, calcification, pleural retraction, and lobulation were independent factors for indeterminate SMSPNs diagnosis. In addition, we identified that 19 key CT-based radiomics features were independent predictors for indeterminate SMSPNs diagnosis. Finally, a nomogram was constructed, and it achieved high efficiency in the preoperative diagnosis of indeterminate SMSPNs, which is of great significance in guiding clinical decision-making.

This study had some potential limitations. First, this work was a retrospective study, and some selective bias may exist. Second, this work was a single-center study and lacked external validation. There were 5 CT scanners in our hospital, and the training cohort and validation cohort were grouped based on different CT scanners. The monogram achieved good results in the training and validation cohorts, indicating that the model had good generalization capability. However, further validation of the nomogram is still needed through larger, prospective studies with more diverse datasets to assess its generalization capability.

In conclusion, this study developed a nomogram incorporating the rad-score, clinical factors, and CT features for the diagnosis of solitary indeterminate SMSPNs. The diagnosis ability of the nomogram was better than that of the radiomics model, clinical model, and experienced radiologists who specialized in thoracic radiology. The nomogram provided a new method for preoperative diagnosis of solitary indeterminate SMSPNs.

## Data availability statement

The raw data supporting the conclusions of this article will be made available by the authors, without undue reservation.

## Ethics statement

This study was reviewed and approved by the Research Ethics Committee of Yantai Yuhuangding Hospital, and patient informed consent was waived.

## Author contributions

CZ: Conceptualization, Data curation, Writing – original draft. HZ: Conceptualization, Investigation, Writing – original draft. ML: Conceptualization, Data curation, Formal analysis, Project administration, Writing – original draft. XY: Data curation, Methodology, Writing – original draft. JL: Data curation, Methodology, Writing – original draft. ZD: Methodology, Software, Writing – review & editing. HM: Conceptualization, Funding acquisition, Supervision, Writing – review & editing. PW: Conceptualization, Funding acquisition, Supervision, Writing – review & editing.

## References

[1] Liao JH Amin VB Kadoch MA Beasley MB Jacobi AH. Subsolid pulmonary nodules: CT–pathologic correlation using the 2011 IASLC/ATS/ERS classification. Clin Imaging. (2015) 39:344–51. doi: 10.1016/j.clinimag.2014.12.009 25709110

[2] Dong H Yin L-K Qiu Y-G Wang X-B Yang J-J Lou C-C. Prediction of high-grade patterns of stage IA lung invasive adenocarcinoma based on high-resolution CT features: a bicentric study. Eur Radiol. (2023) 33:3931–40. doi: 10.1007/s00330-022-09379-x 36600124

[3] Chen B Li Q Hao Q Tan J Yan L Zhu Y. Malignancy risk stratification for solitary pulmonary nodule: A clinical practice guideline. J Evidence-Based Med. (2022) 15:142–51. doi: 10.1111/jebm.12476 35775869

[4] Yang W Sun Y Fang W Qian F Ye J Chen Q. High-resolution computed tomography features distinguishing benign and Malignant lesions manifesting as persistent solitary subsolid nodules. Clin Lung Cancer. (2018) 19:e75–83. doi: 10.1016/j.cllc.2017.05.023 28822623

[5] She Y Zhao L Dai C Ren Y Jiang G Xie H. Development and validation of a nomogram to estimate the pretest probability of cancer in Chinese patients with solid solitary pulmonary nodules: A multi-institutional study. J Surg Oncol. (2017) 116:756–62. doi: 10.1002/jso.24704 28570780

[6] Zhang C-R Wang Q Feng H Cui Y-Z Yu X-B Shi G-F. Computed-tomography-based radiomic nomogram for predicting the risk of indeterminate small (5–20 mm) solid pulmonary nodules. Diagn Intervent Radiol. (2023) 29:283–90. doi: 10.4274/dir.2022.22395 PMC10679690 36987938

[7] Yin J Xi J Liang J Zhan C Jiang W Lin Z. Solid components in the mediastinal window of computed tomography define a distinct subtype of subsolid nodules in clinical stage I lung cancers. Clin Lung Cancer. (2021) 22:324–31. doi: 10.1016/j.cllc.2021.02.015 33789831

[8] Zwirewich CV Vedal S Miller RR Müller NL. Solitary pulmonary nodule: high-resolution CT and radiologic-pathologic correlation. Radiology. (1991) 179:469–76. doi: 10.1148/radiology.179.2.2014294 2014294

[9] Feng B Chen X Chen Y Liu K Li K Liu X. Radiomics nomogram for preoperative differentiation of lung tuberculoma from adenocarcinoma in solitary pulmonary solid nodule. Eur J Radiol. (2020) 128:109022. doi: 10.1016/j.ejrad.2020.109022 32371184

[10] Mankidy BJ Mohammad G Trinh K Ayyappan AP Huang Q Bujarski S. High risk lung nodule: A multidisciplinary approach to diagnosis and management. Respir Med. (2023) 214:107277. doi: 10.1016/j.rmed.2023.107277 37187432

[11] Erasmus JJ Connolly JE McAdams HP Roggli VL. Solitary pulmonary nodules: part I. Morphologic evaluation for differentiation of benign and Malignant lesions. RadioGraphics. (2000) 20:43–58. doi: 10.1148/radiographics.20.1.g00ja0343 10682770

[12] Xu DM van der Zaag-Loonen HJ Oudkerk M Wang Y Vliegenthart R Scholten ET. Smooth or Attached Solid Indeterminate Nodules Detected at Baseline CT Screening in the NELSON Study: Cancer Risk during 1 Year of Follow-up. Radiology. (2009) 250:264–72. doi: 10.1148/radiol.2493070847 18984780

[13] Martin MD Kanne JP Broderick LS Kazerooni EA Meyer CA. RadioGraphics update: lung-RADS 2022. RadioGraphics. (2023) 43:e230037. doi: 10.1148/rg.230037 37856315

[14] Zhu Y Yip R Zhang J Cai Q Sun Q Li P. Radiologic features of nodules attached to the mediastinal or diaphragmatic pleura at low-dose CT for lung cancer screening. Radiology. (2024) 310:e231219. doi: 10.1148/radiol.231219 38165250 PMC10831475

[15] Zhao J Sun L Sun K Wang T Wang B Yang Y. Development and validation of a radiomics nomogram for differentiating pulmonary cryptococcosis and lung adenocarcinoma in solitary pulmonary solid nodule. Front Oncol. (2021) 11:759840. doi: 10.3389/fonc.2021.759840 34858836 PMC8630666

[16] Gillies RJ Kinahan PE Hricak H. Radiomics: images are more than pictures, they are data. Radiology. (2016) 278:563–77. doi: 10.1148/radiol.2015151169 PMC4734157 26579733

[17] Feng B Chen X Chen Y Lu S Liu K Li K. Solitary solid pulmonary nodules: a CT-based deep learning nomogram helps differentiate tuberculosis granulomas from lung adenocarcinomas. Eur Radiol. (2020) 30:6497–507. doi: 10.1007/s00330-020-07024-z 32594210

[18] Zwanenburg A Vallières M Abdalah MA Aerts HJWL Andrearczyk V Apte A. The image biomarker standardization initiative: standardized quantitative radiomics for high-throughput image-based phenotyping. Radiology. (2020) 295:328–38. doi: 10.1148/radiol.2020191145 PMC7193906 32154773

[19] Xiangfei C Panli Z Xunhong Y Fang W Yuwei X Ying C. RadCloud—An artificial intelligence-based research platform integrating machine learning-based radiomics, deep learning, and data management. J Artif Intell Med Sci. (2021) 2:97–102. doi: 10.2991/jaims.d.210617.001

[20] van Griethuysen JJM Fedorov A Parmar C Hosny A Aucoin N Narayan V. Computational radiomics system to decode the radiographic phenotype. Cancer Res. (2017) 77:e104–e7. doi: 10.1158/0008-5472.CAN-17-0339 PMC5672828 29092951

[21] Davis SD. CT evaluation for pulmonary metastases in patients with extrathoracic Malignancy. Radiology. (1991) 180:1–12. doi: 10.1148/radiology.180.1.2052672 2052672

[22] Ha T Kim W Cha J Lee YH Seo HS Park SY. Differentiating pulmonary metastasis from benign lung nodules in thyroid cancer patients using dual-energy CT parameters. Eur Radiol. (2021) 32:1902–11. doi: 10.1007/s00330-021-08278-x 34564746

[23] Siegelman SS Khouri NF Scott WW Leo FP Hamper UM Fishman EK. Pulmonary hamartoma: CT findings. Radiology. (1986) 160:313–7. doi: 10.1148/radiology.160.2.3726106 3726106

[24] Zhang J Han T Ren J Jin C Zhang M Guo Y. Discriminating small-sized (2 cm or less), noncalcified, solitary pulmonary tuberculoma and solid lung adenocarcinoma in tuberculosis-endemic areas. Diagnostics. (2021) 11:930. doi: 10.3390/diagnostics11060930 34064284 PMC8224307

[25] Matsubara T Tagawa T Toyokawa G Kamitani T Takada K Oba T. Radiologic features of resected lung adenocarcinoma with epithelial–mesenchymal transition. Ann Thorac Surg. (2021) 112:1647–55. doi: 10.1016/j.athoracsur.2020.10.034 33248987

[26] Watari N Yamaguchi K Terada H Hamai K Masuda K Nishimura Y. Characteristic computed tomography features in mesenchymal-epithelial transition exon14 skipping-positive non-small cell lung cancer. BMC Pulmon Med. (2022) 22:260. doi: 10.1186/s12890-022-02037-4 PMC9245203 35773658

[27] Park KY Kim SJ Noh TW Cho SH Lee DY Paik HC. Diagnostic efficacy and characteristic feature of MRI in pulmonary hamartoma. J Comput Assist Tomogra. (2008) 32:919–25. doi: 10.1097/RCT.0b013e31815abed4 19204455

[28] Hansen CP Holtveg H Francis D Rasch L Bertelsen S. Pulmonary hamartoma. J Thorac Cardiovasc Surg. (1992) 104:674–8. doi: 10.1016/S0022-5223(19)34735-X 1513155

